# Supplementary material for: Single-cell transcriptional profiling of splenic fibroblasts reveals subset-specific innate immune signatures in homeostasis and during viral infection
Source: Commun Biol. 2021 Dec 2;4:1355. doi: 10.1038/s42003-021-02882-9 (PMC8640036; doi:10.1038/s42003-021-02882-9)
Supplement: Supplementary file 2 — Supplementary Information [file 42003_2021_2882_MOESM2_ESM.pdf]

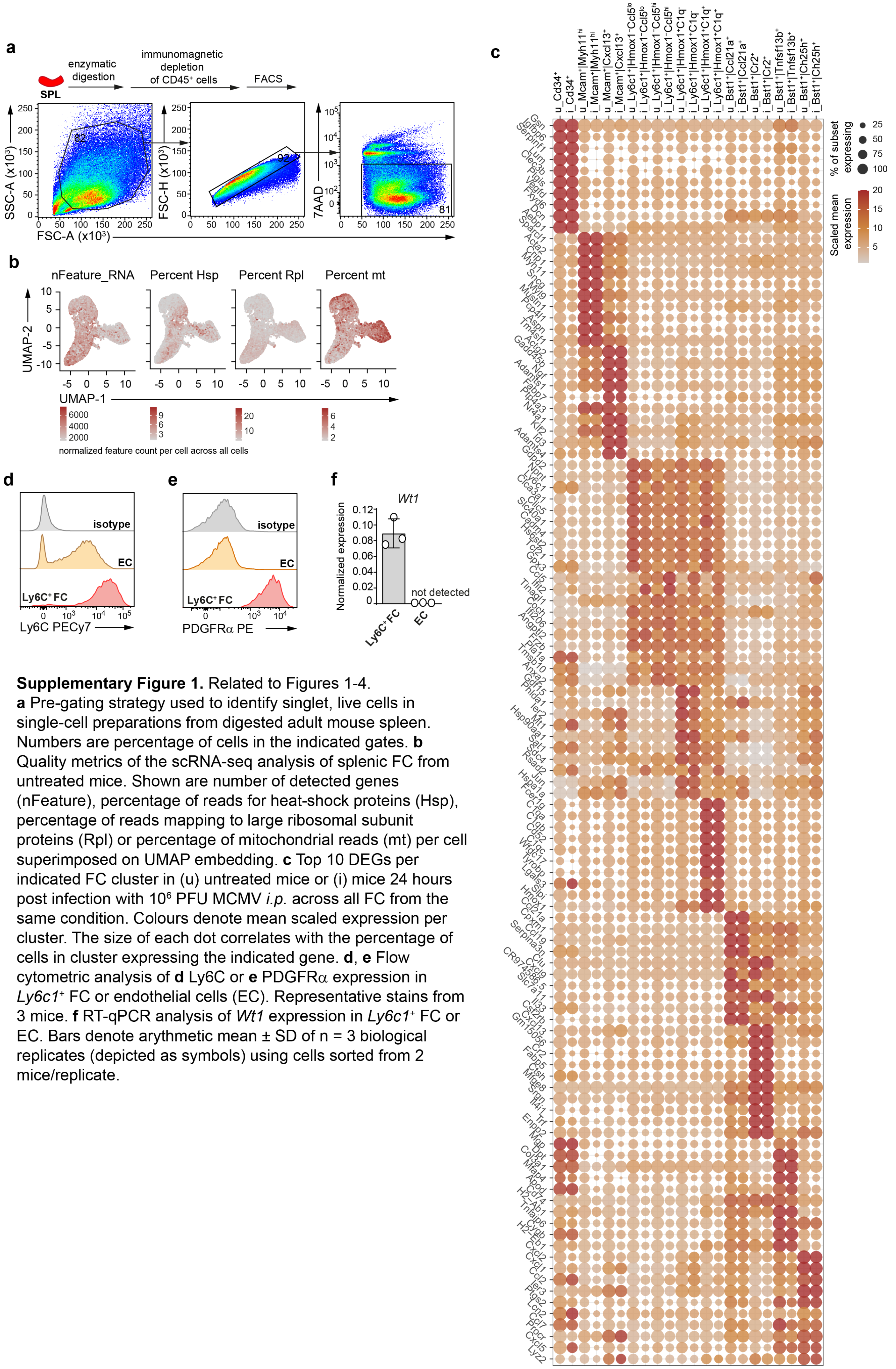

Supplementary Figure 1. Related to Figures 1-4.
a Pre-gating strategy used to identify singlet, live cells in single-cell preparations from digested adult mouse spleen. Numbers are percentage of cells in the indicated gates. **b** Quality metrics of the scRNA-seq analysis of splenic FC from untreated mice. Shown are number of detected genes (nFeature), percentage of reads for heat-shock proteins (Hsp), percentage of reads mapping to large ribosomal subunit proteins (Rpl) or percentage of mitochondrial reads (mt) per cell superimposed on UMAP embedding. **c** Top 10 DEGs per indicated FC cluster in (u) untreated mice or (i) mice 24 hours post infection with 10^6 PFU MCMV *i.p.* across all FC from the same condition. Colours denote mean scaled expression per cluster. The size of each dot correlates with the percentage of cells in cluster expressing the indicated gene. **d**, **e** Flow cytometric analysis of **d** Ly6C or **e** PDGFRα expression in *Ly6c1*⁺ FC or endothelial cells (EC). Representative stains from 3 mice. **f** RT-qPCR analysis of *Wt1* expression in *Ly6c1*⁺ FC or EC. Bars denote arythmetic mean ± SD of n = 3 biological replicates (depicted as symbols) using cells sorted from 2 mice/replicate.

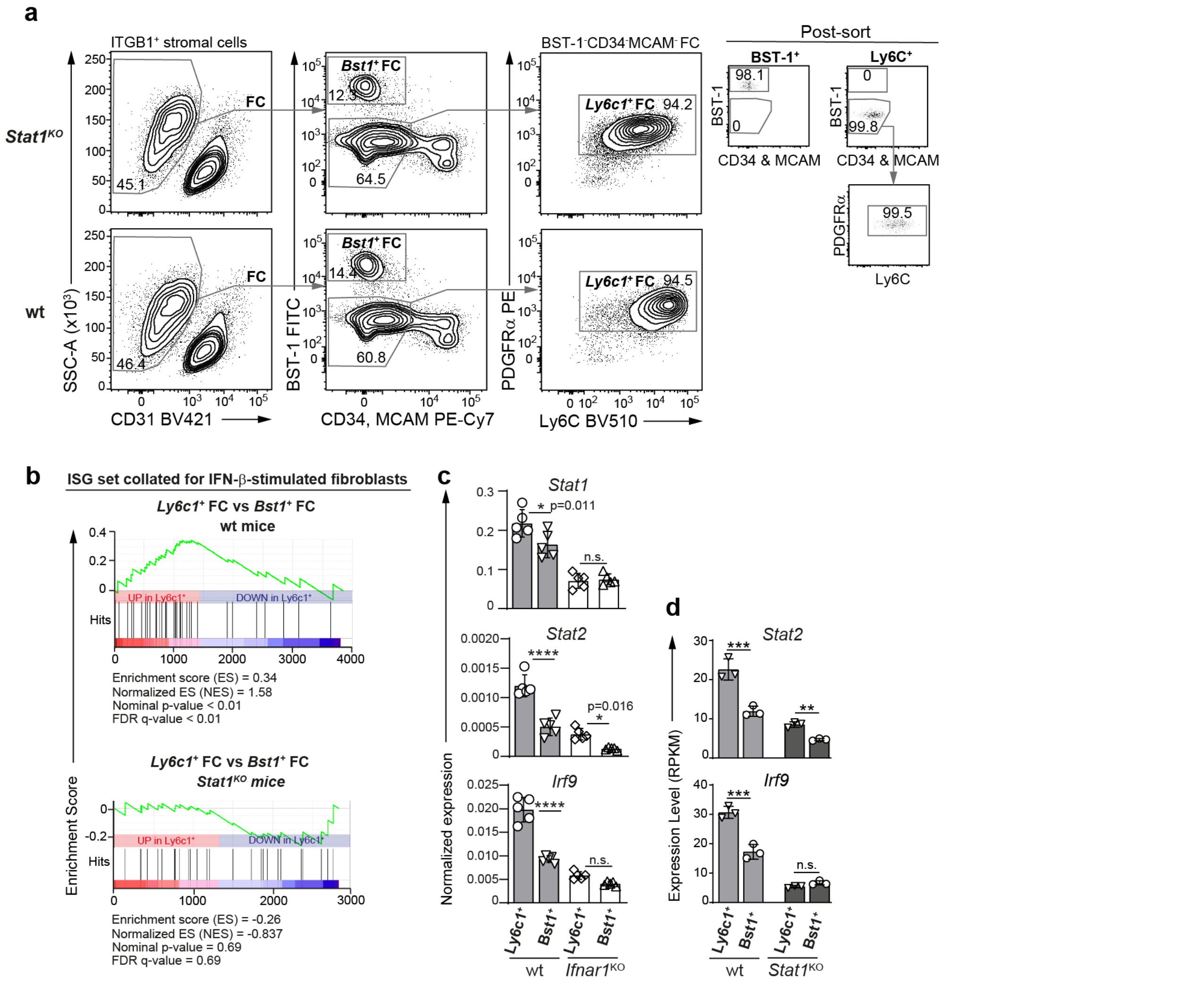

Supplementary Figure 2. Related to Figures 5-6.
a FACS gating on *Ly6c1*⁺ FC and *Bst1*⁺ FC in *Stat1*KO mice. Numbers are percentage of cells in the indicated gates. **b** Gene set enrichment analysis for IFN-β-stimulated gene set collated for primary fibroblasts (extracted from the Interferome database v2.0 using the following search criteria: fold change > 2.5; p-val < 0.05; max. 6 hours post stimulation (http://www.interferome.org)) performed on DEGs ($\log_2$(fold change) > 0.8, adj. p-val < 0.05) from the comparison between *Ly6c1*⁺ FC versus *Bst1*⁺ FC in wt or *Stat1*KO mice. **c** Expression of indicated IFNAR signalling mediators, based on RT-qPCR analysis of *Ly6c1*⁺ FC and *Bst1*⁺ FC isolated from the spleens of wt or *Ifnar1*KO mice. Bars denote mean ± SD of n = 5 mice (depicted as symbols). Statistical significance was calculated using one-way ANOVA with Sidak's multiple comparison test. **d** Expression of indicated IFNAR signalling mediators, based on RNA-seq analysis of *Ly6c1*⁺ FC and *Bst1*⁺ FC isolated from the spleens of wt mice or *Stat1*KO mice. Bars denote mean RPKM ± SD of n = 3 biological replicates (depicted as symbols) using cells sorted from 2-4 mice per replicate. Shown are adjusted p-values from the comparison between *Ly6c1*⁺ FC versus *Bst1*⁺ FC transcriptomes in the wt or the *Stat1*KO condition. **c**, **d** * p<0.05; ** p<0.01; *** p<0.001; **** p<0.0001; n.s. denotes p>0.05

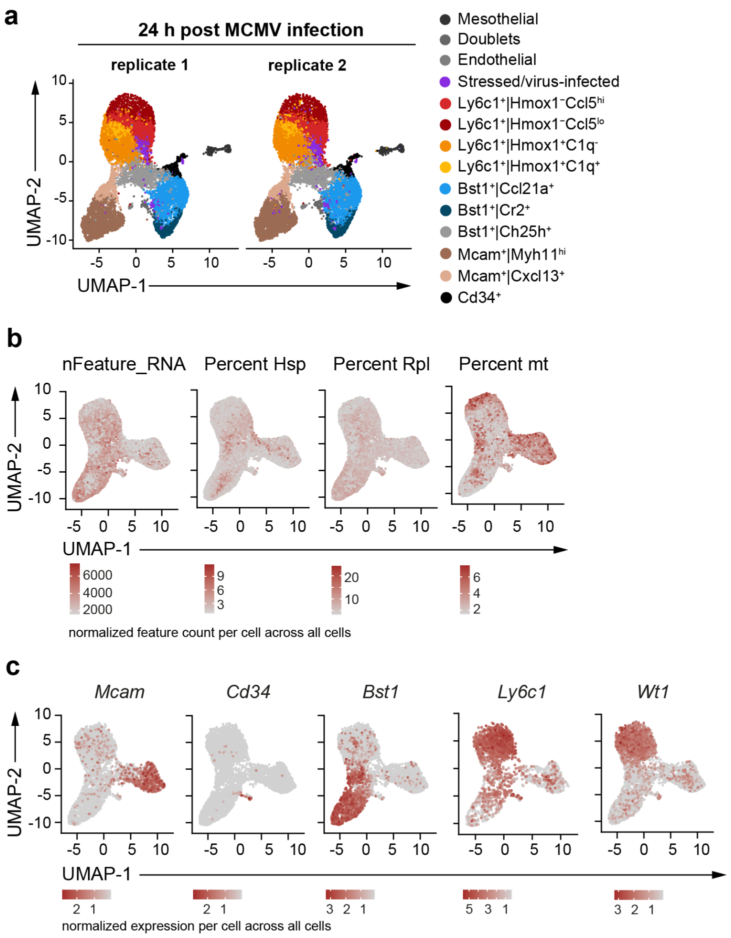

Supplementary Figure 3. Related to Figure 7.
a-**c** scRNA-seq analysis of splenic FC 24 hours post infection with 10^6 PFU of MCMV *i.p.* **a** UMAP embedding before removal of doublets, endothelial-, mesothelial- and virus-infected cells. **b** Number of detected genes (nFeature), percentage of reads for heat-shock proteins (Hsp), percentage of reads mapping to large ribosomal subunit proteins (Rpl) or percentage of mitochondrial reads (mt) per cell superimposed on UMAP embedding. **c** Gene expression on UMAP embedding in the infected condition.

	Wt mice		*Stat1* ^{KO} mice	
**ISGs**	**$\log_2$(fold change) *Ly6c1*$^+$ FC vs *Bst1*$^+$ FC**	**adj. p-val**	**$\log_2$(fold change) *Ly6c1*$^+$ FC vs *Bst1*$^+$ FC**	**adj. p-val**
Rtp4	3.776	6.93E-43	6.285	2.17E-27
Ifit3	3.235	4.69E-28	2.129	4.30E-49
Ifit1	3.119	4.18E-32	1.686	0.001317706
Plac8	2.731	3.25E-22	1.817	4.24E-23
Cmpk2	2.420	8.27E-74	2.381	1.97E-66
Oas1a	2.239	3.14E-22	0.000	1
Isg15	2.165	9.57E-19	-0.329	0.286874755
Gbp3	2.109	2.41E-30	1.724	3.73E-16
Xaf1	2.073	1.96E-20	0.962	1.02E-10
Irf7	1.930	4.69E-16	1.698	1.72E-29
Spats2l	1.892	1.08E-29	1.932	4.79E-11
Rsad2	1.798	1.39E-12	3.469	7.3905E-05
Lgals9	1.790	3.29E-24	0.737	8.28E-18
Tmem140	1.759	5.77E-12	1.542	0.000102696
Zbp1	1.755	4.42E-12	-0.750	0.008939974
Ifitm3	1.577	2.56E-09	0.847	7.93E-21
Gbp4	1.550	7.92E-24	0.396	0.581391389
Rnf213	1.435	1.20E-11	0.456	0.152926136
Ifi44	1.421	4.35E-11	-5.897	0.005285034
Ly6e	1.416	8.48E-14	0.432	4.55343E-11
Ddx60	1.404	1.93E-12	0.356	0.35772351
Ifitm2	1.392	5.10E-08	0.682	5.61E-12
Agrn	1.350	0.003024882	0.806	0.002022399
Parp14	1.325	6.00E-08	1.268	4.76171E-05
Zcchc24	1.295	3.39E-06	1.397	2.60E-38
Mx2	1.293	0.000751404	1.719	0.008415425
Ifit2	1.263	2.20E-08	0.625	0.0581921
Phf11b	1.243	0.000476852	0.669	0.249007739
Trim25	1.213	1.85E-06	0.929	0.001642548
Ifitm1	1.195	0.000276969	0.037	0.821690635
Mlkl	1.173	1.03E-11	0.674	0.051807094
Parp12	1.169	1.93E-28	0.254	0.699968757
Rnf114	1.146	2.32E-18	0.504	3.58415E-05
Usp18	1.137	0.00015923	-0.765	0.025493647
Slfn5	1.077	5.70E-05	1.322	2.35E-14
Phf11d	1.075	1.84E-09	-0.278	0.198582662
Ifi35	1.025	2.25E-05	0.279	0.075168912
Tnfsf10	0.960	0.04825153	0.398	0.679097299
Stat2	0.923	2.60E-09	0.861	0.001569826
Trim21	0.899	2.13E-07	2.980	0.005067653
Ifih1	0.869	1.20E-11	0.222	0.322400559
Eif2ak2	0.844	0.000660729	0.107	0.819661336
Samd9l	0.841	8.66E-10	-0.166	0.601906348
Dhx58	0.832	0.000126611	0.035	0.972571033
Irf9	0.829	2.10E-06	-0.312	0.212880211
Sat1	0.822	1.16E-11	1.558	8.33E-31
Parp10	0.753	5.15E-06	0.930	0.025892372
Cnp	0.703	6.38E-06	0.944	1.31E-08
Parp9	0.683	1.17E-07	-0.107	0.793685179
Atp10a	0.676	0.245699711	0.964	0.564600977
Dtx3l	0.675	0.049529815	0.069	0.936476322
Scarb2	0.674	4.35E-06	0.574	1.99E-14
Fbxo6	0.655	0.005837012	0.433	0.120868253
Gbp2	0.566	7.18E-06	0.500	0.004266245
Zc3hav1	0.527	3.96E-08	0.153	0.147412801
Psme2	0.514	0.05725131	-0.056	0.866395447
Chmp5	0.504	0.000257715	0.269	0.103556815
Adar	0.464	0.244573091	0.036	0.981364291
Acot9	0.458	0.015137002	0.167	0.630351861
Tmem106a	0.446	0.042776103	0.196	0.207427517
Psme1	0.396	0.009505137	-0.027	0.920311469
Gbp6	0.391	0.048587847	-0.691	0.088445103
Gbp5	0.336	0.230637709	0.387	0.378482139
Unc93b1	0.333	0.006382631	-0.196	0.65845475
Myd88	0.321	0.062127335	0.220	0.345129693
Ube2l6	0.270	0.304295277	-0.442	0.000950503
Gsdmd	0.258	0.06256598	0.311	0.158642212
Rab24	0.203	0.467691547	-1.139	0.022104957
Ankfy1	0.182	0.78942167	0.113	0.785947397
Sp140	0.167	0.680294345	0.023	0.971973367
Samhd1	0.156	0.312458174	-0.111	0.536120397
Bst2	0.132	0.577588507	-1.517	3.81E-26
B2m	0.118	0.541853297	-0.975	3.90E-16
Irf1	0.118	0.579921157	1.399	2.70E-19
Lap3	0.079	0.69339683	0.054	0.877140009
Tdrd7	0.077	0.775181231	-0.187	0.726626325
Sp110	-0.024	0.932055876	-0.538	0.066958384
Bag1	-0.026	0.9167317	-0.674	0.135394211
Ddx58	-0.054	0.798735084	-0.590	2.79188E-05
Znfx1	-0.077	0.913316284	-0.222	0.720211365
Lgals3bp	-0.080	0.644637116	-1.911	4.27E-85
Nod1	-0.212	0.368998841	-0.159	0.676973253
Psmb8	-0.219	0.496817363	-0.995	2.00E-31
N4bp1	-0.288	0.245197809	-0.080	0.930491534
Psmb9	-0.387	0.10403244	-0.816	4.10E-10
Nub1	-0.463	2.54E-07	-0.491	0.082969968
Trafd1	-0.729	3.18E-08	-1.200	5.08E-08
Tap1	-0.762	8.75E-10	-1.476	3.75E-28
Ly96	-0.924	3.05E-08	-1.578	2.29E-14
Casp1	-0.978	1.08E-08	-1.011	3.26617E-05
Serpinb9	-1.175	7.71E-24	-1.776	9.1971E-125
Epsti1	-1.191	0.052868151	-0.602	0.719587324
Casp4	-1.266	1.82E-10	-1.676	1.27E-13
Isg20	-1.288	1.43E-06	-2.206	2.51E-31
Stambpl1	-1.551	1.87E-20	-1.243	0.00599388
Vrk2	-1.625	6.18E-26	-1.417	3.44E-06
Lysmd2	-1.836	6.17E-15	-1.635	5.23E-40
Map3k8	-2.089	1.19E-32	-0.859	0.000234561
Abtb2	-2.249	2.74E-13	-1.497	0.26401582
Lcp2	-3.622	3.32E-36	-2.891	1.26925E-09

Supplementary Table 1. Related to Fig. 6

Shown are fold changes in the expression of individual ISGs between *Ly6c1*$^{+}$ FC versus *Bst1*$^{+}$ FC in wt or *Stat1*^{KO} mice. ISGs that are no longer overexpressed in *Ly6c1*$^{+}$ FC upon loss of *Stat1* are highlighted in grey.

Antibody	Source	Cat. No.
APC/Cy7 anti-mouse ITGB1 (HMβ1-1)	BioLegend	Cat# 102226
FITC anti-mouse ITGB1 (HMβ1-1)	BioLegend	Cat# 102205
Biotin anti-mouse ITGB1 (HMβ1-1)	BioLegend	Cat# 102203
Alexa Fluor 700 anti-mouse CD45.2 (104)	BioLegend	Cat# 109822
Brilliant Violet 510 anti-mouse CD45.2 (104)	BioLegend	Cat# 109838
Biotin anti-mouse CD34 (RAM34)	Thermo Fisher	Cat# 13-0341-82
eFluor 660 anti-mouse CD34 (RAM34)	Thermo Fisher	Cat# 50-0341-82
PE/Cy7 anti-mouse MCAM (ME-9F1)	BioLegend	Cat# 134714
Alexa Fluor 647 anti-mouse MCAM (ME-9F1)	BioLegend	Cat# 134718
Purified anti-mouse BST-1 (BP-3)	BioLegend	Cat# 140202
PE anti-mouse BST-1 (BP-3)	BioLegend	Cat# 140204
APC anti-mouse BST-1 (BP-3)	BioLegend	Cat# 140207
FITC anti-mouse BST-1 (KT157)	Thermo Fisher	Cat# MA5-17948
PE anti-mouse PDGFRα (APA5)	BioLegend	Cat# 135906
PE/Cy7 anti-mouse CR2/CR1 (7E9)	BioLegend	Cat# 123419
PE/Cy7 anti-mouse Ly6C (HK1.4)	BioLegend	Cat# 128017
Brilliant Violet 510 anti-mouse Ly6C (HK1.4)	BioLegend	Cat# 128033
Brilliant Violet 785 anti-mouse Ly6C (HK1.4)	BioLegend	Cat# 128041
Biotin anti-mouse Ly-6C (HK1.4)	BioLegend	Cat# 128003
BV421 anti-mouse/human CD45R/B220 (RA3-6B2)	BioLegend	Cat# 103251
APC anti-mouse CD71 (RI7217)	BioLegend	Cat# 113819
BV421 anti-mouse CD31 (MEC13.3)	BD Horizon	Cat# 562939
Biotin anti-mouse Pan-endothelial Cell Antigen (MECA-32)	BioLegend	Cat# 120504
Alexa Fluor 488 anti-mouse Pan-endothelial Cell Antigen Antibody (MECA-32)	BioLegend	Cat# 120506
PE Rat IgG2a Isotype Ctrl (RTK2758)	BioLegend	Cat# 400507
PE/Cy7 Rat IgG2a, κ Isotype Ctrl (RTK2758)	BioLegend	Cat# 400521
APC Rat IgG2a, κ Isotype Ctrl (RTK2758)	BioLegend	Cat# 400511
PE/Cy7 Rat IgG2c, κ Isotype Ctrl (RTK4174)	BioLegend	Cat# 400721
Purified Mouse IgG1, κ Isotype Ctrl Antibody (MOPC-21)	BioLegend	Cat# 400101
Polyclonal anti-mouse CCL21	LifeSpan BioSciences	Cat# LS-C104634-50
FITC anti-alpha smooth muscle actin (1A4)	Abcam	Cat# ab8211
Purified HMOX1 Monoclonal Antibody (HO-1-1)	ThermoFisher	Cat# MA1-112
APC CXCL13 Monoclonal Antibody (DS8CX13)	ThermoFisher	Cat# 17-7981-82
Desmin Polyclonal Antibody	ThermoFisher	Cat# PA5-16705
anti-mouse WT1 (SC06-41)	LIFE Technologies	Cat# MA532215
Rabbit IgG Isotype Control	Thermo Fisher	Cat# 10500C
Goat anti-Rabbit IgG (H+L) Secondary, Alexa Fluor 647	Thermo Fisher	Cat# A-21245
Brilliant Violet 785 Streptavidin	BioLegend	Cat# 405249
PE/Cyanine7 Streptavidin	BioLegend	Cat# 405206

Supplementary Table 2. Related to Methods

Antibodies used in the study.

	Forward (5' - 3')	Reverse (5' - 3')
Gapdh	TGTGTCCGTCGTGGATCTGA	CCTGCTTCACCACCTTCTTGAT
Oas1a	GTCCTGGGTCATGTTAATACTTCCA	GCTCCGTGAAGCAGGTAGAG
Ifit1	GAGCAGAGAGTCAAGGCAGG	CACCATCAGCATTCTCTCCCA
Ifi44	TGGCATTCTGCATTTGGCTT	TCCAGCTTGGACTTCACAGG
Ifih1	TGCTTATCGCTACGACGGTG	TTCATCAGCTCTGGCTCGAC
Eif2ak2	TCGTGACCGGAGTGGAGTAT	GTTGCAAGGCCAAAGTCTCC
Irf7	GGGACCTCTTGCTTCAGGTT	AGGGTTCCTCGTAAACACGG
Usp18	CGGACAGACGTGTTGCCTTA	TCCGAGGCACTGTTATCCTCT
Stat1	AGAGGTGTTGAGTTGGCAGT	ACCATCAGGGCCAGCATTAG
Stat2	CTATTGCTGCCCAAGCTGGA	AACTTTGCTCCAGCCGTCAA
Irf9	AGCAACTGCAACTCTGAGCTA	GTGAGCAGCAGCGAGTAGT
Wt1	CATCCGCAACCAAGGATACAG	TGAAGGAATGGTTGGGGAACT

Supplementary Table 3. Related to Methods

Primer sequences.
