# Supplementary material for: Single-cell transcriptional profiling of splenic fibroblasts reveals subset-specific innate immune signatures in homeostasis and during viral infection
Source: Commun Biol. 2021 Dec 2;4:1355. doi: 10.1038/s42003-021-02882-9 (PMC8640036; doi:10.1038/s42003-021-02882-9)
Supplement: Supplementary file 3 — Description of Additional Supplementary Files [file 42003_2021_2882_MOESM3_ESM.pdf]

Description of Additional Supplementary Files

File name: Supplementary Data 1
Description: Top 100 differentially expressed genes (average log_2(fold change) > 0.25, adjusted p-value < 0.01, fraction of expressing cells 0.1) with the highest fold change per FC cluster across all cells.

File name: Supplementary Data 2
Description: Source data for Figures 3f, 4e, 5e, 6c, 6f, 7a, 7b, 7c, 7i and Supplementary Figures 2c and 2d.
