# Supplementary material for: Single-cell transcriptional profiling of splenic fibroblasts reveals subset-specific innate immune signatures in homeostasis and during viral infection
Source: Commun Biol. 2021 Dec 2;4:1355. doi: 10.1038/s42003-021-02882-9 (PMC8640036; doi:10.1038/s42003-021-02882-9)
Supplement: Supplementary file 6 — Reporting Summary [file 42003_2021_2882_MOESM6_ESM.pdf]

nature research

Corresponding author(s): Katarzyna Sitnik (lead contact), Luka Cicin-Sain

Last updated by author(s): 31 October, 2021

Reporting Summary

Nature Research wishes to improve the reproducibility of the work that we publish. This form provides structure for consistency and transparency in reporting. For further information on Nature Research policies, see our Editorial Policies and the Editorial Policy Checklist.

Statistics

For all statistical analyses, confirm that the following items are present in the figure legend, table legend, main text, or Methods section.

n/a	Confirmed	
☐	☒	The exact sample size (*n*) for each experimental group/condition, given as a discrete number and unit of measurement
☐	☒	A statement on whether measurements were taken from distinct samples or whether the same sample was measured repeatedly
☐	☒	The statistical test(s) used AND whether they are one- or two-sided<br>*Only common tests should be described solely by name; describe more complex techniques in the Methods section.*
☒	☐	A description of all covariates tested
☐	☒	A description of any assumptions or corrections, such as tests of normality and adjustment for multiple comparisons
☐	☒	A full description of the statistical parameters including central tendency (e.g. means) or other basic estimates (e.g. regression coefficient) AND variation (e.g. standard deviation) or associated estimates of uncertainty (e.g. confidence intervals)
☐	☒	For null hypothesis testing, the test statistic (e.g. *F*, *t*, *r*) with confidence intervals, effect sizes, degrees of freedom and *P* value noted<br>*Give P values as exact values whenever suitable.*
☒	☐	For Bayesian analysis, information on the choice of priors and Markov chain Monte Carlo settings
☒	☐	For hierarchical and complex designs, identification of the appropriate level for tests and full reporting of outcomes
☒	☐	Estimates of effect sizes (e.g. Cohen's *d*, Pearson's *r*), indicating how they were calculated

Our web collection on statistics for biologists contains articles on many of the points above.

Software and code

Policy information about availability of computer code

Data collection	No custom computer code or new algorithm were developed in the context of this study.
Data analysis	FlowJo software version 10 (www.flowjo.com)<br>GraphPad Prism versions 8 (www.graphpad.com)<br>ZEN Lite software (www.zeiss.com)<br>Olympus VS-ASW-FL software (www.olympus-lifescience.com)<br>Cell Ranger 3.0.3 (10x Genomics; www.10xgenomics.com)<br>R 3.6.2 (R-project; www.r-project.org)<br>Seurat 3.1.5 (Butler et al., 2018; https://doi.org/10.1038/nbt.4096)<br>topGO (Alexa and Rahnenfuhrer, 2020; https://doi.org/10.18129/B9.bioc.topGO)<br>ggplot2 (CRAN; https://ggplot2.tidyverse.org)<br>GSEA 4.1.0 (https://www.gsea-msigdb.org)<br>STAR (Dobin et al., 2013; https://doi.org/10.1093/bioinformatics/bts635)<br>DESeq2 (Love et al., 2014; https://doi.org/10.1186/s13059-014-0550-8)<br>htseq-count (Anders et al., 2015; https://doi.org/10.1093/bioinformatics/btu638)

For manuscripts utilizing custom algorithms or software that are central to the research but not yet described in published literature, software must be made available to editors and reviewers. We strongly encourage code deposition in a community repository (e.g. GitHub). See the Nature Research guidelines for submitting code & software for further information.

Data

Policy information about availability of data

All manuscripts must include a data availability statement. This statement should provide the following information, where applicable:

- Accession codes, unique identifiers, or web links for publicly available datasets
- A list of figures that have associated raw data
- A description of any restrictions on data availability

RNAseq and scRNAseq data generated during this study will be available at NCBI GEO (GSE156162) after the acceptance of the paper.

Field-specific reporting

Please select the one below that is the best fit for your research. If you are not sure, read the appropriate sections before making your selection.

[x] Life sciences [ ] Behavioural & social sciences [ ] Ecological, evolutionary & environmental sciences

For a reference copy of the document with all sections, see nature.com/documents/nr-reporting-summary-flat.pdf

Life sciences study design

All studies must disclose on these points even when the disclosure is negative.

Sample size	Quantitative analyses (flow cytometry, RT-qPCR) were routinely performed at sample sizes 3-5 biological replicates per group, which were sufficient to generate statistically significant results.
Data exclusions	No data were excluded from the study.
Replication	Results have been confirmed in at least 2 independent experiments.
Randomization	Mice were allocated to groups based on genotyping results.
Blinding	No investigator blinding was used in this study.

Reporting for specific materials, systems and methods

We require information from authors about some types of materials, experimental systems and methods used in many studies. Here, indicate whether each material, system or method listed is relevant to your study. If you are not sure if a list item applies to your research, read the appropriate section before selecting a response.

Materials & experimental systems

n/a	Involved in the study	
[ ]	[x]	Antibodies
[x]	[ ]	Eukaryotic cell lines
[x]	[ ]	Palaeontology and archaeology
[ ]	[x]	Animals and other organisms
[x]	[ ]	Human research participants
[x]	[ ]	Clinical data
[x]	[ ]	Dual use research of concern

Methods

n/a	Involved in the study	
[x]	[ ]	ChIP-seq
[ ]	[x]	Flow cytometry
[x]	[ ]	MRI-based neuroimaging

Antibodies

Antibodies used

APC/Cy7 anti-mouse ITGB1 (HMβ1-1) BioLegend Cat# 102226
FITC anti-mouse ITGB1 (HMβ1-1) BioLegend Cat# 102205
Biotin anti-mouse ITGB1 (HMβ1-1) BioLegend Cat# 102203
Alexa Fluor 700 anti-mouse CD45.2 (104) BioLegend Cat# 109822
Brilliant Violet 510 anti-mouse CD45.2 (104) BioLegend Cat# 109838
Biotin anti-mouse CD34 (RAM34) Thermo Fisher Cat# 13-0341-82
eFluor 660 anti-mouse CD34 (RAM34) Thermo Fisher Cat# 50-0341-82
PE/Cy7 anti-mouse MCAM (ME-9F1) BioLegend Cat# 134714
Alexa Fluor 647 anti-mouse MCAM (ME-9F1) BioLegend Cat# 134718
Purified anti-mouse BST-1 (BP-3) BioLegend Cat# 140202
PE anti-mouse BST-1 (BP-3) BioLegend Cat# 140204
APC anti-mouse BST-1 (BP-3) BioLegend Cat# 140207

FITC anti-mouse BST-1 (KT157) Thermo Fisher Cat# MA5-17948
PE anti-mouse PDGFRa (APA5) BioLegend Cat# 135906
PE/Cy7 anti-mouse CR2/CR1 (7E9) BioLegend Cat# 123419
PE/Cy7 anti-mouse Ly6C (HK1.4) BioLegend Cat# 128017
Brilliant Violet 510 anti-mouse Ly6C (HK1.4) BioLegend Cat# 128033
Brilliant Violet 785 anti-mouse Ly6C (HK1.4) BioLegend Cat# 128041
Biotin anti-mouse Ly-6C (HK1.4) BioLegend Cat# 128003
BV421 anti-mouse/human CD45R/B220 (RA3-6B2) BioLegend Cat# 103251
APC anti-mouse CD71 (RI7217) BioLegend Cat# 113819
BV421 anti-mouse CD31 (MEC13.3) BD Horizon Cat# 562939
Biotin anti-mouse Pan-endothelial Cell Antigen (MECA-32) BioLegend Cat# 120504
Alexa Fluor 488 anti-mouse Pan-endothelial Cell Antigen Antibody (MECA-32) BioLegend Cat# 120506
PE Rat IgG2a Isotype Ctrl (RTK2758) BioLegend Cat# 400507
PE/Cy7 Rat IgG2a, κ Isotype Ctrl (RTK2758) BioLegend Cat# 400521
APC Rat IgG2a, κ Isotype Ctrl (RTK2758) BioLegend Cat# 400511
PE/Cy7 Rat IgG2c, κ Isotype Ctrl (RTK4174) BioLegend Cat# 400721
Purified Mouse IgG1, κ Isotype Ctrl Antibody (MOPC-21) BioLegend Cat# 400101
Polyclonal anti-mouse CCL21 LifeSpan
BioSciences Cat# LS-C104634-50
FITC anti-alpha smooth muscle actin (1A4) Abcam Cat# ab8211
Purified HMOX1 Monoclonal Antibody (HO-1-1) ThermoFisher Cat# MA1-112
APC CXCL13 Monoclonal Antibody (DS8CX13) ThermoFisher Cat# 17-7981-82
Desmin Polyclonal Antibody ThermoFisher Cat# PA5-16705
anti-mouse WT1 (SC06-41) LIFE Technologies Cat# MA532215
Rabbit IgG Isotype Control Thermo Fisher Cat# 10500C
Goat anti-Rabbit IgG (H+L) Secondary, Alexa Fluor 647 Thermo Fisher Cat# A-21245
Brilliant Violet 785 Streptavidin BioLegend Cat# 405249
PE/Cyanine7 Streptavidin BioLegend Cat# 405206

Validation

Antibodies used in this study were selected based on relevant validation statements on the manufacturer's website (i.e. detection of mouse antigen as well as application in flow cytometry or confocal microscopy).

Animals and other organisms

Policy information about studies involving animals; ARRIVE guidelines recommended for reporting animal research

Laboratory animals

In the experiment addressing fibroblast populations during spleen development (Fig. 1f), 3-d old mice or 6-w old mice on C57BL/6 background were used. All other experiments were performed with age- and sex-matched 2-5 m old mice on C57BL/6 background. C57BL/6JrJ mice were purchased from Janvier Labs. Ifnar1-KO, Stat1-KO and PdgfraH2B-GFP mice (described before, see Methods section) were bred and maintained under specific pathogen free, SPF conditions according to Federation of European Laboratory Animal Science Associations (FELASA) guidelines respectively at central animal facility of HZI Braunschweig, Germany; University of Veterinary Medicine Vienna, Austria and San Raffaele Scientific Institute, Milan, Italy.

Wild animals

The study did not involve wild animals.

Field-collected samples

The study did not involve samples collected from the field.

Ethics oversight

Animal procedures were approved by the responsible state office (Lower Saxony State Office of Consumer Protection and Food Safety, LAVES).

Note that full information on the approval of the study protocol must also be provided in the manuscript.

Flow Cytometry

Plots

Confirm that:

- [x] The axis labels state the marker and fluorochrome used (e.g. CD4-FITC).
- [x] The axis scales are clearly visible. Include numbers along axes only for bottom left plot of group (a 'group' is an analysis of identical markers).
- [x] All plots are contour plots with outliers or pseudocolor plots.
- [x] A numerical value for number of cells or percentage (with statistics) is provided.

Methodology

Sample preparation

Spleens were digested with collagenase P (0.4 mg/ml), dispase II (2 mg/ml) and DNase I (50 µg/ml) in RPMI-1640 supplemented with 1 mM sodium pyruvate, 100 U/ml penicillin, 100 U/ml streptomycin, 10 mM HEPES and 5 % fetal bovine serum (FBS). Spleens were pre-incubated with the above digestion solution injected into the organ using a 26G needle for 5 min at RT, then minced using scissors and digested for 30 min at 37 °C. Enzymatic treatment was repeated for additional 20

April 2020

	min followed by incubation with 5 mM EDTA for 5 min at RT. The resulting cell suspensions were passed through a 70 µm filter, followed by immunomagnetic depletion of CD45+ cells using MACS (Miltenyi Biotech). Briefly, cells from one spleen were incubated with 60 µl anti-CD45 microbeads in 600 µl of PBS containing 2 mM EDTA and 2 % FBS for 20 min on ice, washed, and depleted on LS columns according to manufacturer's instructions (Miltenyi Biotech).
Instrument	Data acquisition was performed on an Aria-II SORP, ARIA-Fusion or LSR-Fortessa (BD Biosciences). Sorting was performed on an Aria-II SORP or ARIA-Fusion (BD Biosciences).
Software	FlowJo software 10 (BD)
Cell population abundance	Sorting purities were routinely determined by flow cytometric re-analysis of sorted cell populations. Samples showing ≥ 95% purity of sorted cells were qualified for downstream analysis.
Gating strategy	All flow cytometric analyses were performed following exclusion of dead cells (identified using 7-AAD Viability Staining Solution or Zombie NIR Fixable Viability Kit; both from BioLegend) and cell aggregates (identified on FSC-A versus FSC-H scatter plots). Stromal cells were identified as live, singlet, CD45-, ITGB1+ cells. Stromal cells were further split into CD31+ endothelial cells (EC) and CD31- fibroblastic cells (FC). Gating strategy is shown in Fig. 1a and Supplemental Fig. 1a.

☒ Tick this box to confirm that a figure exemplifying the gating strategy is provided in the Supplementary Information.
